# Revisiting Quantification of Phenylalanine/Tyrosine Flux in the Ochronotic Pathway during Long-Term Nitisinone Treatment of Alkaptonuria

**DOI:** 10.3390/metabo12100920

**Published:** 2022-09-29

**Authors:** Lakshminarayan R. Ranganath, Andrew T. Hughes, Andrew S. Davison, Milad Khedr, Richard Imrich, Mattias Rudebeck, Birgitta Olsson, Brendan P. Norman, George Bou-Gharios, James A. Gallagher, Anna M. Milan

**Affiliations:** 1 Departments of Clinical Biochemistry and Metabolic Medicine, Liverpool University Hospitals NHS Foundation Trusts, Liverpool L7 8XP, UK; 2 Department of Musculoskeletal & Ageing Science, Institute of Life Course & Medical Sciences, University of Liverpool, Liverpool L69 3BX, UK; 3 National Institute of Rheumatic Diseases, Biomedical Research Centre, Slovak Academy of Sciences, 22095 Bratislava, Slovakia; 4 Biomedical Research Centre, Slovak Academy of Sciences, 831 01 Bratislava, Slovakia; 5 OnPoint Science AB, 111 29 Stockholm, Sweden; 6 Garriguella AB, 179 62 Ekerö, Sweden

**Keywords:** alkaptonuria, pigment, biliary excretion, ochronosis, nitisinone, homogentisic acid

## Abstract

Changes in the phenylalanine (PHE)/tyrosine (TYR) pathway metabolites before and during homogentisic acid (HGA)-lowering by nitisinone in the Suitability of Nitisinone in Alkaptonuria (AKU) 2 (SONIA 2) study enabled the magnitude of the flux in the pathway to be examined. SONIA 2 was a 48-month randomised, open-label, evaluator-blinded, parallel-group study performed in the UK, France and Slovakia recruiting patients with confirmed AKU to receive either 10 mg nitisinone or no treatment. Site visits were performed at 3 months and yearly thereafter. Results from history, photographs of eyes/ears, whole body scintigraphy, echocardiography and abdomen/pelvis ultrasonography were combined to produce the Alkaptonuria Severity Score Index (cAKUSSI). PHE, TYR, hydroxyphenylpyruvate (HPPA), hydroxyphenyllactate (HPLA) and HGA metabolites were analysed by liquid chromatography/tandem mass spectrometry in 24 h urine and serum samples collected before and during nitisinone. Serum metabolites were corrected for total body water (TBW), and the sum of 24 h urine plus total body water metabolites of PHE, TYR, HPPA, HPLA and HGA were determined. The sum of urine metabolites (PHE, TYR, HPPA, HPLA and HGA) were similar pre- and peri-nitisinone. The sum of TBW metabolites and sum TBW + URINE metabolites were significantly higher peri-nitisinone (*p* < 0.001 for both) compared with pre-nitisinone baseline. Significantly higher concentrations of metabolites from the tyrosine metabolic pathway were observed during treatment with nitisinone. Arguments for unmasking of the ochronotic pathway and biliary elimination of HGA are put forward.

## 1. Introduction

The ochronotic pigment is central to the pathogenesis of alkaptonuria (AKU) [1]. Virchow first coined the term ochronosis when he described a blue/black pigment in the hip and knee joints of a man of 67 years in 1866 at necropsy, in which the cartilage appeared similar to brown ochre under the microscope [2], even though Virchow did not recognise that ochronosis was a feature of AKU. In 1902, Albrecht who showed that alkaptonuria and ochronosis were different components of the same disease [3]. Ochronosis is the key pathophysiological event in AKU and is due to deposition of HGA-pigment in tissues [4]. It is now known that AKU (OMIM 203500) is a condition characterised by the absence of homogentisate 1,2 dioxygenase (HGD) enzyme (EC:1.13.11.5) (Figure S1) leading to accumulation of HGA [5]. As an intermediary in the phenylalanine (PHE)/tyrosine (TYR) pathway, HGA is normally completely metabolised to yield fumarate and acetoacetate [6]. Therefore, in non-AKU subjects, there is no Increase in circulating HGA and little in urine [7]. 

In AKU, the circulating HGA remains increased despite efficient urine excretion [6]. The deposition of circulating HGA as ochronotic HGA-pigment in body tissues leads to rigidity and brittleness, causing these tissues to break down under everyday stresses [8]. Almost all serious co-morbidities of AKU, such as premature arthritis, cardiac valve disease, osteoporosis, fractures and muscle, ligament and tendon ruptures are secondary to tissue ochronosis [6,9,10]. Models of ochronosis, in vitro and in mouse, have confirmed the dose relationships between HGA and ochronosis and their modulation by lowering HGA [11,12]. Ochronosis has only been shown to be partially reversible in areas where it can be externally observed and assessed, such as in the eyes and ears during HGA-lowering [13]. Nothing is known about change in internal ochronosis, such as in spine, joints and tendons, when HGA is decreased. 

Little is known about the ochronotic pathway—the pathway that leads to the formation of pigment from the accumulating HGA. The PHE/TYR pathway reclaims the excess PHE/TYR consumed in the diet so that there is no wastage while ensuring no abnormal increases in these amino acids or their intermediates (Figure S1). There is an equimolar relationship among the PHE/TYR pathway metabolites. In AKU, the pathway that leads to the conversion of accumulating HGA to ochronotic pigment via oxidation products is the most crucial. It is clear that HGA, after being formed, can either be excreted in the urine, remain elevated in total body water (TBW) or be tissue-bound either as HGA or as ochronotic pigment. A recent stable isotope study showed that small molecules of the PHE/TYR pathway distribute not just into the plasma compartment but into whole body water [14]. Reducing the formation of HGA should decrease urinary HGA, TBW HGA as well as HGA-oxidation products. Decreasing HGA is also expected to decrease flux down the ochronotic pigment pathway.

Recently, a group of investigators showed that a small molecule enzyme inhibitor, nitisinone (2-(2-nitro-4-(trifluoromethyl)benzoyl)cyclohexane-1,3-dione) (NIT), decreased serum and urine HGA and slowed progression of AKU disease in the setting of real-world usage in the United Kingdom National Alkaptonuria Centre and in a randomised nitisinone intervention study (Suitability of Nitisinone in Alkaptonuria 2; SONIA 2) [15,16]. Both studies, employing different doses of NIT, showed decreased ochronosis in the eyes and ears, which did not reverse fully. In AKU mice, NIT has been shown to decrease circulating HGA and to inhibit ochronosis [11,12]. NIT inhibits the enzyme p-hydroxyphenylpyruvate dioxygenase (HPPD) in the PHE/TYR pathway, thereby directly leading to a decrease in formation of HGA. 

An earlier short-term study (SONIA 1) analysing the impact of NIT on the ochronotic pathway showed significantly greater amounts of PHE/TYR pathway metabolites during NIT compared with prior to NIT and concluded that it was due to unmasking of the ochronotic pathway [17]. The earlier SONIA 1 study also concluded that approximately half of the daily HGA produced was eliminated in the urine and the other half retained in the body, with the latter being revealed only by inhibiting the production of HGA using NIT [17]. Assays for the measurement of PHE, TYR, 4-hydroxyphenylpyruvate (HPPA), 4-hydroxyphenyllactate (HPLA) and HGA were used in the SONIA 1 analysis, both in serum and urine, and adapted to derive TBW and summed daily metabolites [14,18]. 

The aim of the current data analysis is to assess whether the findings from the 4-week SONIA 1 study of the NIT effect on the ochronotic pathway are supported by similar findings during the analyses of the 48-month SONIA 2 study of NIT in AKU [15].

## 2. Materials and Methods

SONIA 2 was a 48-month, open-label, evaluator-blinded, multicentre, randomised, no-treatment controlled, parallel-group study aiming to recruit 140 patients aged 25 years or older, with a confirmed diagnosis of AKU and any clinical manifestation in addition to increased HGA [15]. Liverpool (UK), Paris (France) and Piešťany (Slovakia) were the three investigational sites, with the Independent Ethics Committee at each centre approving the study. All patients provided a written informed consent prior to inclusion. 

In total, 70 patients were to be randomised to oral NIT 10 mg (Orfadin^®^) daily and 70 to a control (no-treatment) group. NIT was withdrawn in patients who developed signs of ocular tyrosine-related adverse events. If feasible, once the ocular symptoms had resolved (minimum 2 months after temporary withdrawal), NIT was reintroduced at a lower dose (2 mg daily). Alternatively, the patient was withdrawn from the study. If ocular tyrosine-related symptoms reappeared on the lower dose, NIT was permanently withdrawn, and the patient was monitored until the symptoms resolved. More details of the design of the study, including various procedures and assessments, have been previously published [15]. A recommendation, reinforced at each visit, was made to patients receiving NIT to decrease dietary protein consumption to minimise the risk of ocular adverse events, without any further active intervention.

### 2.1. Procedures

The assessments and investigations included the collection of medical history and physical examination, including those specific for AKU, and a wide range of clinical outcome measures, including range of motion tests and quality of life assessments, safety assessment and other procedures shown in Table S1 and elsewhere [15]. The severity of AKU was determined by the AKU Severity Score Index (AKUSSI). The AKUSSI incorporates multiple, clinically meaningful AKU outcomes that can be described in a single score [15]. All items included in the AKUSSI were assessed at baseline and yearly thereafter. Two types of AKUSSI were included as secondary outcomes in SONIA 2. These are the clinical evaluation AKUSSI (cAKUSSI) and a modified AKUSSI (mAKUSSI) (mAKUSSI was cAKUSSI without pigmentation scores). The sum of ear and eye pigment scores as shown in Table S1 gives the ochronosis scores (OCH scores).

Patients visited the study sites at baseline (V1), 3 months (V2) and then annually up to month 48 (V3–V6); a close-out phone call took place at month 49. A physical examination of the patients, including measurement of height and weight, was carried out at each visit. At each visit, 24 h urine was collected into 2.5 L bottles containing 30 mL of 5 N H_2_SO_4_ and stored away from direct sunlight, and PHE/TYR metabolites were assayed by liquid chromatography/tandem mass spectrometry. The weight of the collected urine was recorded and used as the volume in the calculations of amounts of PHE/TYR metabolites, assuming a density of 1 g/mL. An aliquot of the collected urine was frozen and kept at −80 °C until analysis. Blood samples were collected in plain serum tubes (Sarstedt, Germany). An aliquot of serum was immediately acidified using perchloric acid (10% *v*/*v* 5.8 M) to stabilise the HGA and kept frozen at −80 °C until analysis. Samples from Piešťany and Paris were transported frozen to Liverpool, and all biochemical analyses were performed in the Department of Clinical Biochemistry, Liverpool Clinical Laboratories, Liverpool University Hospital NHS Trust.

### 2.2. Chemical Analyses

Measurement of NIT, HGA, TYR, PHE, HPPA and HPLA in serum (indicated as sNIT, sHGA, sTYR, sPHE, sHPPA and sHPLA), as well as a combined analysis (sMETAB) including sHGA, sTYR, sPHE, sHPPA and sHPLA, were carried out on all samples collected at the described sampling points. Further, 24 h urine measurements of HGA, TYR, PHE, HPPA and HPLA (indicated as uHGA24, uTYR24, uPHE24, uHPPA24 and uHPLA24), as well as a combined analysis (uMETAB24) including uHGA24, uTYR24, uPHE24, uHPPA24 and uHPLA24, were also carried out on the collected samples. 

The concentrations of as sNIT, sHGA, sTYR, sPHE, sHPPA, sHPLA and sMETAB (the sMETAB included sPHE, sTYR, sHPPA, sHPLA and sHGA), as well as uHGA24, uTYR24, uPHE24, uHPPA24, uHPLA24 and uMETAB24 (the uMETAB24 included uPHE24, uTYR24, uHPPA24, uHPLA24 and uHGA24), were measured by liquid chromatography/tandem mass spectrometry based on previously published methods [19,20]. In addition, the fully validated methodologies are UKAS 15189-accredited. 

TBW metabolites of PHE, TYR, HPPA, HPLA and HGA (TBW PHE, TBW TYR, TBW HPPA, TBW HPLA, TBW HGA and TBW METAB (the TBW METAB included TBW PHE, TBW TYR, TBW HPPA, TBW HPLA and TBW HGA)): Since PHE and TYR and their metabolites are small molecules that are distributed in TBW [21,22], the concentrations of circulating metabolites were multiplied by the factor of 0.6 times body weight in kg to derive the amounts of TBW metabolites [21,22]. 

Total urinary metabolites: Amounts of the urinary TYR and PHE and their metabolites were similarly calculated by multiplying urine concentrations with the 24 h urine volumes.

Summed metabolites (TBW + 24-h URINE): The TBW and 24 h urine metabolites were added to derive the summed metabolites (SUM HGA, SUM TYR, SUM PHE, SUM HPPA and SUM HPLA and SUM METAB (SUM METAB included SUM HGA, SUM TYR, SUM PHE, SUM HPPA and SUM HPLA). 

### 2.3. Statistical Analysis

Data from those patients who completed study visits and were found to comply with the study procedures were included. Both 2 mg and 10 mg dose data were part of the data analyses of TYR/PHE pathway metabolites provided compliance was satisfactory as evidenced by sNIT (appropriately increased in the NIT group and absent in the control group) and uHGA24 (appropriately decreased in the NIT group and increased in the control group).

All statistical analyses were post-hoc. Continuous variables are presented using mean and standard deviation (SD). All visits were analysed with regard to metabolite relationships both by ANOVA (Tukey-Kramer for multiple comparisons) and simple linear regression. Analyses were performed using Graphpad InStat 3 software (version number 3.1); *p* values < 0.05 were considered statistically significant. 

## 3. Results

At baseline, there were 69 patients in both the control and the NIT groups instead of the planned 70 patients in each group. The mean ± SD age of the control and the NIT groups at baseline were 47.6 ± 10.1 and 49.0 ± 11.3 years, respectively. The mean ± SD body weight of the control and the NIT groups at baseline were similar at 74.1 ± 10.1 and 74.8 ± 14.8 years, respectively. There was no difference in the mean ± SD of the SUM METAB (expressed as µmol) at baseline between the control (42716 ± 15212) and NIT (42268 ± 14177) groups, respectively. There was a totally different pattern in terms of change in SUM METAB (%) (calculated as SUM METAB at V2 minus SUM METAB at V1 divided by SUM METAB at V1 and expressed as %; all other visits V3–V6 were similarly compared against V1) in the NIT group compared to the control (Table 1). At baseline, the mean ± SD cAKUSSI was higher in the NIT group (86.9 ± 34.6) compared to the control (80.4 ± 33.3). Likewise, the mean ± SD mAKUSSI was higher at baseline in the NIT group (56.6 ± 27.2) compared to the control (54.0 ± 24.9). The mean ± SD of the OCH scores in the NIT and control groups at baseline were similar at 21.4 ± 10.7 and 18.0 ± 10.9, respectively (Table 1).

The serum values are not discussed here further but are shown in the supplementary file (Table S2). TBW HGA and TBW PHE showed a significant difference between visits in the control group, whereas the rest of the TBW measures showed no significant differences across visits. In the NIT group, all TBW measurements were significantly different across visits, with TBW HGA decreasing across visits, while all other measurements in TBW increased across visits (Table S3). 

In the control group, the uTYR24 and uPHE24 showed a statistically significant decrease across visits, whereas the uHPLA24 increased across visits, and the rest of the 24 h urine measures showed no significant change. In the NIT group, all 24 h urine measurements were significantly different across visits, with uHGA24 and uPHE24 decreasing across visits, while all other measurements increased across visits (Table S4). 

In the control group, the SUM PHE and SUM HPLA showed a statistically significant increase across visits, whereas the rest of the showed no significant change. In the NIT group, all the individual components of the SUM METAB measurements were significantly different across visits, with HGA decreasing across visits, while all other measurements in the 24 h urine increased across visits (Table S5). 

The molecular weight of HGA of 168 g/mol was used to derive HGA-equivalents, with PHE, TYR, HPPA and HPLA all considered molar equivalents of HGA. Since PHE, TYR and HPPA can give rise to HGA during sequential enzymatic action in the PHE/TYR pathway, on a 1-1 basis, PHE, TYR and HPPA can be considered HGA molar equivalents. When using only urine HGA to calculate the daily burden, this amounts to 6.1 g/day. When using SUM METAB (PHE, TYR, HPPA, HPLA and HGA) to calculate the daily burden in steady state, this amounts to 7.1 g/day prior to NIT administration. During NIT administration, the SUM METAB (PHE, TYR, HPPA, HPLA and HGA) revealed the daily burden in steady state to be 15.32 g/day. The change in HGA-equivalents during NIT administration was an additional 8.21 g/day or 53.6% of the peri-NIT daily burden; this 8.21 g/day was present but hidden before NIT administration. Assuming this daily hidden load of 8.21 g/day remains constant, the yearly HGA-equivalent accumulation within the body amounts to 3.0 kg or 30 kg over 10 years or 210 kg over 70 years in the untreated AKU patient (Table 2). 

Regression analyses between variables (Table S6, Figures S2–S5) in the control group showed a weak statistically significant effect of age on change in SUM METAB (R of 0.12, *p* < 0.05), and a stronger relationship between age and cAKUSSI, mAKUSSI and OCH scores (R of 0.68, 0.59 and 0.22, respectively, *p* < 0.0001 for all). The change in SUM METAB showed a significant relationship with SUM METAB (R of 0.39, *p* < 0.0001) and OCH scores (R of 0.19, *p* < 0.0001). cAKUSSI, mAKUSSI and OCH scores showed strong significant interrelationships (Table S6A). In the female group receiving NIT, regression relationships were similar to male group as shown in Table S6B,C. However, the change in SUM METAB was significantly related to cAKUSSI, mAKUSSI and, more importantly, OCH scores in males and not in females. 

The comparison between the female and male subgroups showed higher SUM METAB, cAKUSSI and mAKUSSI in males both in the control and NIT groups. Age was similar in males and females in the control group, but females were older than males in the NIT group (Table S7). 

**Figure 1:**
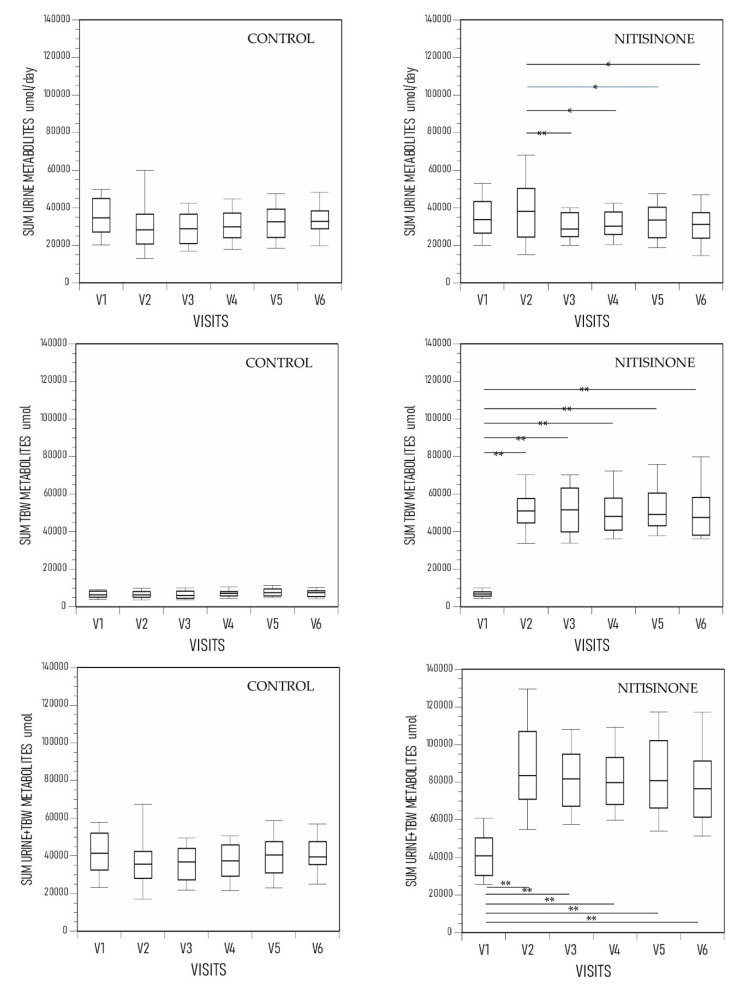
The SUM URINE METAB, SUM TBW METAB and SUM METAB in the control and the nitisinone group of the SONIA 2. (*p* values only indicated for between-visit comparisons where statistical significance was achieved.) The visits range from V1 (baseline), V2 (month 3), V3 (month 12), V4 (month 24), V5 (month 36) to V6 (month 48) (data shown as boxplots with median) (statistical significance p expressed * <0.05, ** <0.01, and *** <0.001 respectively).

The SUM URINE metabolites were similar across visits V1 to V6 in the control group. In the NIT group, the SUM URINE metabolites were significantly higher at V2 compared with subsequent visits (Figure 1). The SUM TBW metabolites were similar across visits V1 to V6 in the control group. In the NIT group, the SUM TBW metabolites were significantly lower at V1 compared with all subsequent visits (Figure 1). 

The SUM METAB was similar across visits V1 to V6 in the control group. In the NIT group, the SUM METAB metabolites were significantly lower at V1 visit compared with all subsequent visits (Figure 1). 

**Figure 2:**
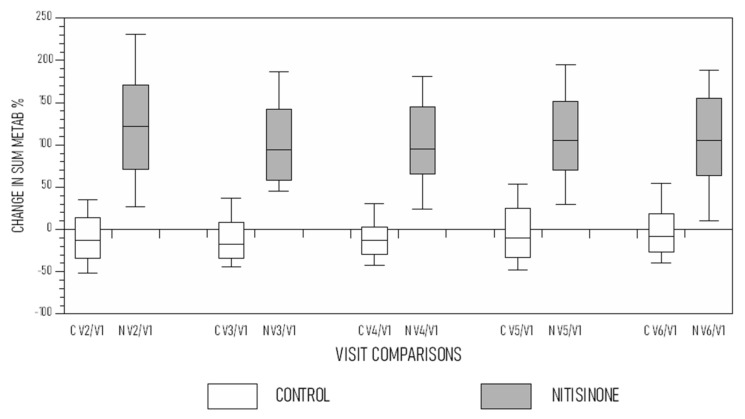
Comparison of change in METAB (%) at V2–V6 compared with V1 ([(V2−V1/V1) × 100] showing stable mostly neutral values in the control group and significantly higher values in the nitisinone group (C V2/V1 refers to comparison of SUM METAB at V2 vs. V1, and so on for other visits along the *X*-axis) (data shown as boxplots with median).

The change in SUM METAB (%) was noted from visit V2 onward compared with V1. For example, comparing visit V2 with V1, the calculation shown in brackets [(V2−V1/V1) × 100] was mostly negative in the control group (Figure 2), and similar calculations were made comparing V3, V4, V5 and V6 against V1. In the NIT group, the change in SUM METAB noted from visit V2–V6 onward compared with V1 [(V2−V1/V1) × 100] was consistently around 100% (Figure 2).

## 4. Discussion

The SONIA 1 study previously examined PHE/TYR pathway metabolites prior to and during nitisinone treatment and found a lack of overall change in urinary metabolites, but a marked increase in TBW metabolites with a near-doubling of SUM METAB during NIT therapy [23]. There was a near 100% increase in the appearance of new PHE/TYR pathway metabolites, namely the change in SUM METAB (%) during NIT therapy compared to baseline pre-NIT values. It was suggested that the newly uncovered metabolites represented unmasking the flux that would otherwise be directed down the ochronotic HGA-pigment pathway in untreated AKU. The previous SONIA 1 was a short-term 4-week study, whereas the current manuscript describes and discusses data from the much larger and longer SONIA 2 study of NIT therapy in AKU. The SONIA 2 provided a further opportunity to rigorously examine the previous suggestion that the appearance of new metabolites during NIT occurs and that this could reflect hidden flux in the ochronotic HGA-pigment pathway. 

The current SONIA 2 data analysis also showed that urine metabolites for the PHE/TYR pathway were similar prior to and during NIT therapy over the 48-month period in the NIT group, while it was similarly stable in the untreated control group over the 48 months. Similar to the SONIA 1 study, the analysis of the urinary metabolites in the SONIA 2 study showed that in the NIT-treated group HGA was the dominant metabolite pre-NIT at baseline, whereas during NIT, HPPA and HPLA replaced HGA as the dominant urinary metabolites. The TBW metabolites in the control group were stable over the 48 months despite showing minor fluctuations consistent with dietary variations over the study duration of 48 months. The NIT group, on the other hand, showed a marked increase in TBW metabolites at all visits from V2 to V6 compared to the baseline visit, with the main reason for the increase in TBW metabolites being increases in TYR with a smaller contribution from HPPA and HPLA (Table S3). As a result, the SUM METAB in the NIT group in the SONIA 2 approximately doubled from V2 to V6 compared with baseline. The changes in SUM METAB mainly reflects TBW changes in TYR with minor contributions from HPPA and HPLA (Table S5).

The appearance of new metabolites reflected by the CHANGE SUM METAB (%) was around 100% in the NIT group in the current study, which persisted at each visit over the 4 years and matched what was found in the earlier SONIA 1 study. We attempted to understand what the appearance of the new metabolites could signify in the pathogenesis of AKU. If, as was suggested in SONIA 1, all the new metabolites represented the flux down the ochronotic HGA-pigment pathway, then this could amount to 8.21 g daily or 2997 g per year or 30 kg over 10 years (Table 2). For an AKU patient at 70 years, assuming constant accretion of HGA-pigment at the same rate over the lifetime, this could amount to more than 200 kg of pigment, assuming that the pigment is irreversible or minimally reversible in the untreated state, which seems implausible. In fact, the amount of pigment seen at post-mortem in a 74-year-old woman who had never received NIT during her life was localised to certain areas such that the amount of pigment observed did not come close to the calculations made here [24]. 

The nature of the ochronotic pigment has come under scrutiny recently. Zannoni suggested more than 50 years ago that an enzyme HGA polyphenol oxidase identified in mammalian skin and cartilage could catalyse the oxidation of HGA to an ochronotic pigment via benzoquinone acetic acid (BQA) and proposed a pathway for the oxidation of HGA via benzoquinone acetic acid to polymeric ochronotic pigment in the tissues of alkaptonuric individuals (Figure S1B) [25]. Recent studies have suggested alternative mechanisms for the pigmentation of tissues, such as radical formation, and have questioned whether the ochronotic pigment is a multimer of HGA [26]. 

The literature is sparse on the ochronotic pigment pathway, but two previous studies have attempted to modulate the ochronotic pathway selectively without affecting the main PHE/TYR pathway. One of these studies suggested that high-dose ascorbic acid decreased the binding of 14C HGA in AKU connective tissues, thus paving the way for ascorbic acid therapy in AKU [27], which has since shown to be largely ineffective [6]. The other study, employing high doses of ascorbic acid in AKU, showed that BQA decreased in bodily fluids without any change in urinary HGA. The authors concluded that this was relevant to ochronosis and was evidence of HGA flux down the ochronotic pathway [28]. The decreased conversion of HGA to BQA was attributed to the antioxidant effect of ascorbic acid. 

The SONIA 2 study data showed a similar quantity of new metabolites during NIT therapy to what was found during the SONIA 1 analysis. The thinking around the SONIA 1 data analysis considered that HGA might bind to tissue or pre-formed pigment loosely, which, over time, might dissociate and return to the circulating pool to be eliminated; therefore, it was reasoned at the time that the appearance of new metabolites might not be sustained over time. This supposition was based on the idea that the ochronotic pigment could reverse at least partially as has been shown during long-term NIT therapy [13,15,16]. However, the result of the SONIA 2 confirms the sustained appearance of new metabolites and suggests that the origin was unlikely to be solely due to ochronotic pigment pathway flux for reasons already discussed.

**Figure 3:**
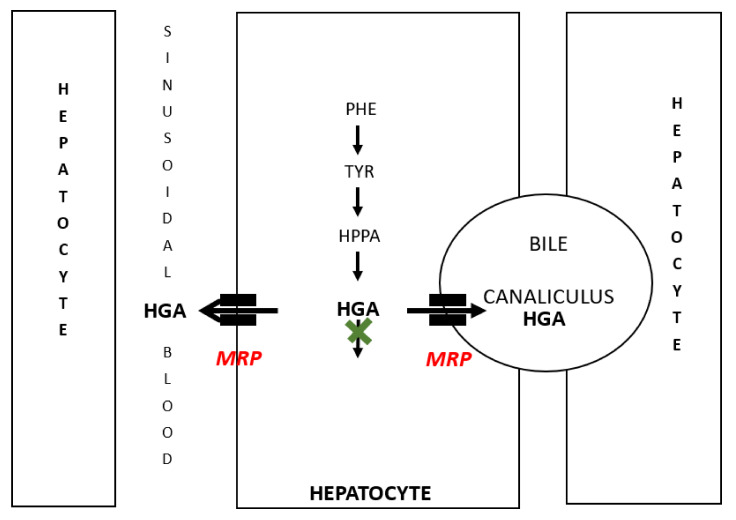
The cartoon depicts the handling of organic anion HGA in the hepatocyte. HGA accumulates due to lack of HGD enzyme (denoted by the green X). We propose that HGA can be eliminated via multidrug resistance-associated proteins (MRP, ABCC family of transporters) both into the biliary canaliculi as well as into the hepatic sinusoidal blood supported by the presence of these organic anion transporters at both these sites. HGA reaching the hepatic sinusoidal blood can be carried around the body before excretion by the kidney into the urine. PHE, TYR, HPPA and HGA represent phenylalanine, tyrosine, 4-hydroxyphenylpyruvate and homogentisate.

**Figure 4:**
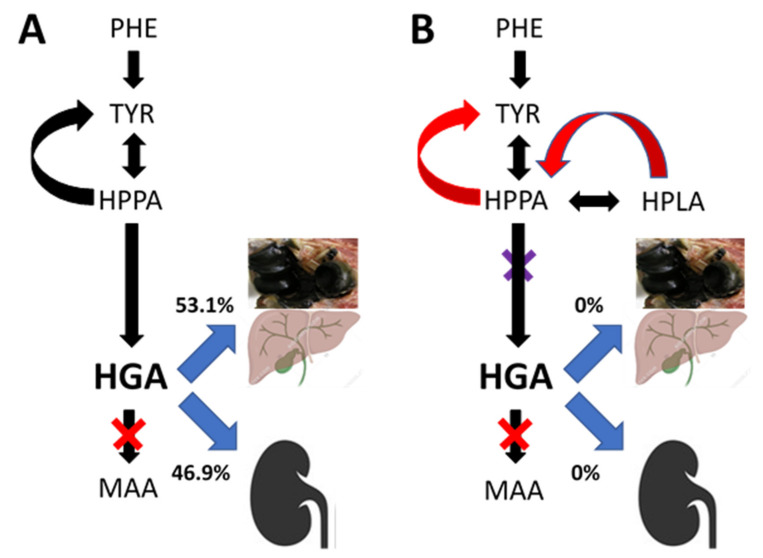
Diagrammatic representation showing that HGA accumulates in AKU due to a deficiency of homogentisate dioxygenase (denoted by the red X), with a measured spill-over into urine as well as predicted biliary excretion and accumulation in the body as pigment. In AKU (Panel (**A**)), circulating HGA is in low concentration relative to urinary excretion and therefore the urinary excretion is dominant. However, data during nitisinone (reflecting 4-hydroxyphenylpyruvate dioxygenase inhibition denoted by the purple) show that 53.1% of TYR flows into the non-urinary pathways including biliary and pigment pathway and only 46.9% is excreted in the urine, following 10 mg nitisinone daily for 4 weeks. Panel (**B**) shows the complete inhibition of HGA urinary and biliary excretion as well as HGA deposition and the suggested mechanism for metabolite generation during nitisinone treatment. (PA: phenylalanine; TYR: tyrosine; HPPA: 4-hydroxyphenylpyruvate; HPLA: 4-hydroxyphenyllactate; HGA: homogentisic acid; MAA: maleylacetoacetic acid) (Image of pigmented elbow joint denotes the total body ochronotic process; that of the liver denotes the biliary excretion, while the kidney image denotes urinary HGA excretion.)

When the hypothesis proposed following the SONIA 1 study (which lasted only 4 weeks) was re-examined in the current SONIA 2 study, the only plausible alternate explanation was that there was excretion of HGA via the biliary tract with elimination via the alimentary tract; this explanation can only work if the biliary HGA was not reabsorbed back into the body but eliminated via the intestine. Our revised hypothesis is shown in Figure 3 and Figure 4. In terms of support for this hypothesis, there are data suggesting that the mechanisms for the export of organic anions exist not only at the sinusoidal surface of the hepatocyte allowing hepatocyte HGA to reach the systemic circulation but also at the sinusoidal surface allowing transfer of hepatic HGA into the bile [29,30,31]. In fact, the MRP2 transporter hypothesised to transfer HGA from the renal proximal tubular cell into the urine is also present in the biliary canalicular surface of the hepatocyte [29,30,31,32]. Nothing has been described about the biliary excretion of HGA in the literature apart from a case report from Denmark [33].

This Danish publication reports that a 50-year-old woman with classic signs of AKU, including discoloration of ear cartilage, dark urine and joint pain, had 30 years of recurrent bile duct stones after having had a cholecystectomy for gallstone-related pain. Despite sufficient papillotomy to minimise stone formation, she had removal of bile duct stones several times during the 30 years following the cholecystectomy. The patient was then started on a low PHE/TYR diet after which her recurrent abdominal pain attacks as well as biliary stone formation stopped immediately, and she had apparently been symptom-free for the 5 years until publication of the case. Unfortunately, the authors of the case report did not provide photographs of the bile duct stones or measure HGA in the bile, but the remission following the low PHE/TYR diet is strongly supportive of their hypothesis. Their case is the first putative demonstration of biliary excretion of HGA [33]. 

Our updated hypothesis for the appearance of substantial amounts of new metabolites during NIT therapy is shown in Figure 4. Approximately 46% of HGA is eliminated in the urine, whereas the balance 54% is partly converted to ochronotic HGA-pigment and partly excreted via the biliary route into the gastrointestinal tract for elimination. The data provide no insight into the proportions of HGA fluxing into the pigment or secreted into bile; however, it is plausible that the major component is the biliary secretion. 

There was no relationship between SUM METAB and age but there was a weak positive effect on change in SUM METAB (%) and age, suggesting that more new metabolites appeared during NIT in older study participants. The direct strong relationship between age and cAKUSSI, mAKUSSI and OCH scores in SONIA 2 at baseline is in line with what we know about the evolution of AKU over the human lifecycle [10,34]. Significantly, the change in SUM METAB (%) showed a positive albeit weak relationship with OCH scores, suggesting that when more metabolites are detected during NIT, these are possibly being converted to ochronotic pigment, especially in males. The weak relationship is consistent with our proposition that the majority of the new metabolites seen during nitisinone therapy were excreted in the bile into the intestine for elimination. The interrelationship between cAKUSSI, mAKUSSI and OCH scores in the SONIA 2 data was strongly positive and confirm that ochronosis underlies severity of AKU. 

There were no major differences during regression analyses between male and female genders. The greater SUM METAB in males is probably due to higher protein intake in this group. The higher cAKUSSI and mAKUSSI in males compared to females is similar to what has been noted previously about the symptomatic disease being more severe and appearing earlier in males [10,34]. The fact that females in the NIT group were older is also consistent with lower severity and a more delayed onset of severity in this group.

There were limitations with the presented analyses. The calculations of TBW employed by us made no allowance for gender and age differences. A single blood sample was used to derive TBW metabolites, although it is known that metabolite concentrations can vary across the 24 h period; however, a recent study showed that fasting metabolite concentrations were within 10% of the 24 h mean values [35]. There is no direct data on the biliary excretion which will require further investigation.

## 5. Conclusions

In conclusion, the amounts of new metabolites appearing during NIT therapy was reconfirmed and found to be sustained over a 48-month period. Calculations of the amount of pigment that would be formed in untreated AKU patients show that the extra metabolites unmasked by NIT therapy could not be mainly due to a flux down the ochronotic pathway. Instead, we propose that secretion of HGA into bile followed by intestinal elimination is the more plausible explanation for the massive increase in metabolites during NIT, although it is likely that a minor proportion of the peri-NIT increases in metabolites could be due to blocking further HGA-pigment formation. Further investigations are needed to clarify the suggested hypothesis.

## Figures and Tables

**Table 1:** Details of SUM METAB (TBW + URINE) in control (n = 69) and nitisinone-treated (n = 69) groups in the combined female and male group.

	Age (Years)	Weight (kg)	SUM METAB (µmol/L)	Change SUM METAB %	cAKUSSI	mAKUSSI	OCH SC
Control group							
V1	47.6 ± 10.1	74.1 ± 15.5	42,716 ± 15,212		80.4 ± 33.3 ^†^	54.0 ± 24.9 *	18.0 ± 10.9
V2	48.1 ± 10.0	74.7 ± 15.6	38,829 ± 19,257	−9.1 ± 36.5			
V3	48.8 ± 10.0	74.3 ± 15.6	36,485 ± 12,065	−9.0 ± 37.0	79.2 ± 34.8	54.8 ± 25.6	17.8 ± 10.9
V4	49.8 ± 10.1	76.0 ± 15.7	37,503 ± 10,428	−11.8 ± 28.2	83.1 ± 33.9	57.2 ± 26.1	17.8 ± 10.7
V5	50.6 ± 10.1	74.9 ± 14.6	40,699 ± 13,268	0.9 ± 47.3	90.1 ± 37.2	63.4 ± 30.3	19.4 ± 10.7
V6	51.6 ± 10.1	73.8 ± 15.0	41,020 ± 11,724	0.2 ± 38.6	95.7 ± 36.4	67.0 ± 29.9	20.3 ± 11.2
Nitisinone group							
V1	49.0 ± 11.3	74.8 ± 14.8	42,268 ± 14,177 ****		86.9 ± 34.6	56.6 ± 27.2	21.4 ± 10.7
V2	49.2 ± 11.3	75.7 ± 15.1	91,145 ± 29,629	131 ± 106			
V3	49.8 ± 11.3	78.1 ± 15.0	82,204 ± 20,743	110 ± 86	84.2 ± 34.0	57.2 ± 27.3	20.9 ± 11.1
V4	50.8 ± 11.3	78.6 ± 15.3	82,343 ± 21,724	110 ± 85	91.1 ± 35.6	62.1 ± 28.3	21.1 ± 10.6
V5	51.3 ± 11.3	78.6 ± 15.9	84,610 ± 22,801	111 ± 64	88.8 ± 38.8	61.2 ± 31.1	20.2 ± 10.8
V6	52.6 ± 11.4	78.4 ± 16.1	80,874 ± 24,976	105 ± 65	94.8 ± 37.3	66.4 ± 31.2	20.3 ± 10.8

V1–V6 refers to visits to study sites at baseline, 3, 12, 24, 36 and 48 months; values are shown as Mean ± SD; statistical comparisons within control or nitisinone group denotes whether there were differences within the group across visits and to what degree indicated as: ^†^—*p* = 0.058; * <0.05; ** <0.01; *** <0.001; **** <0.0001.

**Table 2:** Estimates of HGA * equivalents generated pre- and peri-nitisinone to calculate burden of accumulating HGA over time.

uHGA_24_	36,361 umol or 6.1 g per day
All HGA equivalents pre-NIT in TBW and 24-h urine	42,268 umol or 7.1 g per day or 46.4% total daily PHE/TYR flux
All HGA equivalents during NIT	91,145 umol or 15.32 g per day
Additional HGA equivalents during NIT	8.21 g or 53.6% total daily PHE/TYR flux per day
Additional HGA equivalents during NIT per year (365 days)	2997 g or 3.0 kg per year
Additional HGA equivalents during NIT per 10 years	30 kg per 10 years

* HGA MW 168 g/mol used in calculation; PHE/TYR/HPPA/HPLA and HGA all considered molar equivalents in these calculations.

## Data Availability

SONIA 2 data access will be granted in response to qualified research requests. All de-identified individual participant data, for patients with separate consent signed for this purpose, can be made available to researchers. Data will be shared based on the scientific merit of the proposal—i.e., the proposal should be scientifically sound, ethical and have the potential to contribute to the advancement of public health as well as the feasibility of the research proposal—i.e., the requesting research team must be scientifically qualified and have the resources to conduct the proposed project. The data files would exclude data dictionaries that require user licenses. Data could be made available following finalized regulatory authority review and end of any data exclusivity periods and ending after 36 months or until the corresponding author is able to fulfil this obligation, whichever is earlier. Further, the study protocol and statistical analysis plan can be made available. Proposals should be directed to j.a.gallagher@liverpool.ac.uk to gain access. Data requestors will need to sign a data access agreement.

## Supplementary Materials

The following supporting information can be downloaded at: https://www.mdpi.com/article/10.3390/metabo12100920/s1, Figure S1A. The phenylalanine/tyrosine pathway; Figure S2. Simple linear regression relationships between (A) Age (years) vs. SUM METAB umol, (B) Age (years) vs. CHANGE SUM METAB %, (C) Age (years) vs. cAKUSSI and (D) Age (years) vs. mAKUSSI (Linear regression coefficients shown as R with degree of statistical significance shown as Asterix); Figure S3 Simple linear regression relationships between (A) SUM METAB (µmol) vs. CHANGE SUM METAB %, (B) SUM METAB (µmol) vs. cAKUSSI, (C) SUM METAB (µmol) vs. mAKUSSI and (D) SUM METAB (µmol) vs. OCH SCORES (Linear regression coefficients shown as R with degree of statistical significance shown as Asterix); Figure S4. Simple linear regression relationships between (A) CHANGE SUM METAB % vs. cAKUSSI, (B) CHANGE SUM METAB % vs. mAKUSSI, (C) CHANGE SUM METAB % vs. OCH SCORES (Linear regression coefficients shown as R with degree of statistical significance shown as Asterix); Figure S5. Simple linear regression relationships between (A) cAKUSSI vs. mAKUSSI, (B) cAKUSSI vs. OCH SCORES, and (C) mAKUSSI vs. OCH SCORES (Linear regression coefficients shown as R with degree of statistical significance shown as Asterix). Table S1. Clinical AKU Severity Score Index (cAKUSSI) (procedure shown in italics); Table S2. Serum metabolites in control (n = 69) and nitisinone-treated (n = 69) groups in combined female and male group; Table S3. TBW metabolites in control (n = 69) and nitisinone-treated (n = 69) groups in combined female and male group; Table S4. 24-h urine metabolites in control (n = 69) and nitisinone-treated (n = 69) groups in combined female and male group; Table S5. SUM METAB in control (n = 69) and nitisinone-treated (n = 69) groups in combined female and male group; Table S6A. Regression relationships between variables in the control group. S6B. Regression relationships between variables in the NIT group (Females). S6C. Regression relationships between variables in the NIT group (Males) (Linear regression coefficients shown are R with degree of statistical significance shown in Asterix); Table S7. Comparison of variables between the female and male subgroups in the control (n = 441) and nitisinone groups (n = 374) (Linear regression coefficients shown are R with degree of statistical significance shown in Asterix).
