# Supplementary material for: Revisiting Quantification of Phenylalanine/Tyrosine Flux in the Ochronotic Pathway during Long-Term Nitisinone Treatment of Alkaptonuria
Source: Metabolites. 2022 Sep 29;12(10):920. doi: 10.3390/metabo12100920 (PMC9610527; doi:10.3390/metabo12100920)
Supplement: Supplementary file 1 [file metabolites-12-00920-s001.zip › metabolites-1895886-supplementary.pdf]

Supplementary file

Legend to Supplementary tables and figure

Supplementary Tables

Table S1. Clinical AKU Severity Score Index (cAKUSSI) (*procedure shown in italics*)

Table S2. Serum metabolites in control (n=69) and nitisinone-treated (n=69) groups in combined female and male group

Table S3. TBW metabolites in control (n=69) and nitisinone-treated (n=69) groups in combined female and male group

Table S4. 24-h urine metabolites in control (n=69) and nitisinone-treated (n=69) groups in combined female and male group

Table S5. SUM METAB in control (n=69) and nitisinone-treated (n=69) groups in combined female and male group.

Table S6A. Regression relationships between variables in the control group. S6B. Regression relationships between variables in the NIT group (Females). S6C. Regression relationships between variables in the NIT group (Males) (Linear regression coefficients shown are R with degree of statistical significance shown in Asterix).

Table S7. Comparison of variables between the female and male subgroups in the control (n = 441) and nitisinone groups (n = 374) (Linear regression coefficients shown are R with degree of statistical significance shown in Asterix).

Supplementary figure

Figure S1A. The phenylalanine/tyrosine pathway. The PHE/TYR metabolic pathway is shown highlighting the site of the enzyme defect observed in AKU and the site of action of nitisinone, a reversible competitive inhibitor of 4-hydroxyphenylpyruvate dioxygenase. The pathway also highlights the dynamic relationships between HPPA, TYR and HPLA, a key relationship after introduction of nitisinone. Figure S1B. The ochronotic pathway proposed by Zannoni et al (REF 26) is shown diagrammatically. The accumulating HGA in AKU is oxidised to benzoquinone acetate (BQA) which then polymerises to ochronotic pigment. Zannoni et al proposed that this was catalysed by a specific HGA-polyphenol oxidase. Ascorbic acid and N-acetylcysteine has been proposed as antioxidants to inhibit the conversion of HGA to BQA. They further proposed that HGA, BQA and the polymeric BQA bind to connective tissue macromolecules through physical and chemical bonds further fixing the ochronotic pigment. The red cross refers to lack of homogentisate 1,2 dioxygenase enzyme in AKU.

Figure S2. Simple linear regression relationships between (A) Age (years) vs SUM METAB umol, (B) Age (years) vs CHANGE SUM METAB %, (C) Age (years) vs cAKUSSI and (D) Age (years) vs mAKUSSI (Linear regression coefficients shown as R with degree of statistical significance shown as Asterix).

Figure S3 Simple linear regression relationships between (A) SUM METAB (µmol) vs CHANGE SUM METAB %, (B) SUM METAB (µmol) vs cAKUSSI, (C) SUM METAB (µmol) vs mAKUSSI and (D) SUM METAB (µmol) vs OCH SCORES (Linear regression coefficients shown as R with degree of statistical significance shown as Asterix).

Figure S4. Simple linear regression relationships between (A) CHANGE SUM METAB % vs cAKUSSI, (B) CHANGE SUM METAB % vs mAKUSSI, (C) CHANGE SUM METAB % vs OCH SCORES (Linear regression coefficients shown as R with degree of statistical significance shown as Asterix)

Figure S5. Simple linear regression relationships between (A) cAKUSSI vs mAKUSSI, (B) cAKUSSI vs OCH SCORES, and (C) mAKUSSI vs OCH SCORES (Linear regression coefficients shown as R with degree of statistical significance shown as Asterix).

Table S1.

Table S1. Clinical AKU Severity Score Index (cAKUSSI)(*mAKUSSI) (*procedure shown in italics*)					
Feature		Score	Feature		Score
CLINICAL FEATURES (excluding spine and joint)					
Eye pigment *(Standardised Medical Photography)*					
Right eye (Nasal)	Slight	4	Left eye (Nasal)	Slight	4
	Marked	8		Marked	8
Right eye (Temporal)	Slight	4	Left eye (Temporal)	Slight	4
	Marked	8		Marked	8
Ear pigment *(Standardised Medical Photography)*					
Right ear	Slight	2	Left ear	Slight	2
	Marked	4		Marked	4
Stones *(Ultrasonography of abdomen and pelvis)*					
Prostate Stones	Per episode	4	Renal Stones	Per episode	4
Musculoskeletal					
Bone mineral density of hip<br>*Dual Energy X-Ray Absorbtiometry (DEXA)*	Grade (T-scores)<br>≥ -1·0<br>-1·0 to -1·7<br>-1·8 to -2·4<br>≤ -2·5	 0<br>2<br>4<br>6			
Adult fracture<br>*(Questionnaire)*	Per fracture	8	Ligament rupture	Per rupture	8
Tendon rupture<br>*(Questionnaire)*	Per rupture	8	Muscle rupture	Per rupture	8
Heart *(Transthoracic echocardiography)*					
Normal<br>Aortic sclerosis		0<br>4	Aortic valve stenosis	Mild<br>Moderate<br>Severe	8<br>10<br>12
ENT *(Audiometry)*					
Hearing impairment	Grade on audiometry (dB loss), per ear<br>≤ 20<br>21-35 (mild)<br>36-60 (moderate)<br>>60 (severe)	 0<br>1<br>2<br>4	Dark tympanic membrane<br>*(Otoscopic examination)*	Per ear	6
JOINT FEATURES					
Clinical joint pain (1 for each large joint area; hips, knees, ankles, feet, shoulders, elbows, wrists & hands - right and left sides = 14 joint areas) *(Questionnaire)*					Max 14
Non-spine joint disease (2 for each large joint area; hips, knees, ankles, feet, shoulders, elbows, wrists & hands - right and left sides = 14 joint areas) *(either Technetium 99m-methyl diphosphonate or* ^{18F}*PETCT)*					Max 28
Arthroscopies *(Questionnaire)*					2 each
Joint replacements *(Questionnaire)*					4 each
SPINE FEATURES					
Clinical spinal pain (2 each for cervical, thoracic, lumbar, sacroiliac) *(Questionnaire)*					Max 8
Spine disease (4 each for pubic symphysis, ribs, sacroiliac, lumbar, thoracic, cervical)<br>*(either Technetium 99m-methyl diphosphonate or* ^{18F}*PETCT)*					Max 24

Table S1. Clinical AKU Severity Score Index (cAKUSSI)(*mAKUSSI) (*procedure shown in italics*)					
Feature		Score	Feature		Score
Kyphosis	(Cobb angles)		Scoliosis	(Cobb angles)	
(X-Ray Lateral Spine and pelvis)	<45	0	*X-Ray antero-posterior Spine and pelvis*	<5	0
	45-60	3		5-20	2
	>60	6		21-30	4
				>30	6
*mAKUSSI refers to cAKUSSI minus eye+ear pigment scores					

Table S2.

Table S2. Serum metabolites in control (n=69) and nitisinone-treated (n=69) groups in combined female and male group						
	sHGA µmol/L	sTYR µmol/L	sPHE µmol/L	sHPPA µmol/L	sHPLA µmol/L	sNIT µmol/L
Control group (n=69)						
V1	28.3 ± 8.7	64.5 ± 15.5	56.5 ± 9.5			
V2	28.3 ± 9.6	65.2 ± 21.1	56.2 ± 9.8			
V3	28.9 ± 13	62.5 ± 21	54.5 ± 10.8			
V4	32.3 ± 10.9	65 ± 14.4	62.1 ± 9.9			
V5	40.2 ± 18.6	67 ± 14.1	64.5 ± 8.8			
V6	37.1 ± 21	63.5 ± 16.2	65.3 ± 11.9			
Nitisinone group (n=69)						
V1	30.3 ± 11	65.3 ± 14.8	56.8 ± 9.5			
V2	0.7 ± 1.3	951 ± 215	58.8 ± 12.1	40.8 ± 32.2	89.7 ± 29.4	4.2 ± 1.8
V3	0.7 ± 1.6	915 ± 204	58.4 ± 11.7	36.2 ± 7.6	87.4 ± 27	4.3 ± 1.9
V4	0.9 ± 1.1	896 ± 207	64.4 ± 11	39.4 ± 9.6	90.7 ± 33.2	5 ± 2.7
V5	0.9 ± 0.8	939 ± 216	67 ± 12	40.2 ± 11.9	90.6 ± 31.6	5.3 ± 2.7
V6	1.1 ± 1.6	892 ± 288	69 ± 19.5	41.1 ± 15.2	96.4 ± 40.9	6 ± 3.9
V1 – V6 refers to visits to study sites at baseline, 3, 12, 24, 36 and 48 months<br>Values are shown as Mean ± SD						

Table S3.

Table S3. TBW metabolites in control (n=69) and nitisinone-treated (n=69) groups in combined female and male group						
	TBW HGA µmol	TBW TYR µmol	TBW PHE µmol	TBW HPPA µmol	TBW HPLA µmol	SUM TBW METAB µmol
Control group (n=69)						
V1	1266 ± 497****	2948 ± 1196	2557 ± 875**			6770 ± 2339
V2	1282 ± 545	2954 ± 1379	2533 ± 734			6773 ± 2399
V3	1322 ± 755	2873 ± 1419	2474 ± 898			6686 ± 2863
V4	1446 ± 601	2985 ± 1099	2840 ± 931			7271 ± 2357
V5	1818 ± 874	3055 ± 1033	2914 ± 785			7797 ± 2350
V6	1669 ± 1009	2883 ± 1135	2949 ± 965			7501 ± 2678
Nitisinone group (n=69)						
V1	1376 ± 586****	2963 ± 940****	2574 ± 746****			6913 ± 1969****
V2	33.6 ± 61.8	43385 ± 13225	2713 ± 855	1818 ± 1388****	4063 ± 1555****	52012 ± 15365
V3	35.0 ± 80.6	42973 ± 12968	2759 ± 804	1706 ± 526	4099 ± 1377	51572 ± 15012
V4	43.5 ± 48.6	42478 ± 12881	3074 ± 960	1864 ± 599	4311 ± 1751	51770 ± 15245
V5	42.4 ± 39.2	43834 ± 12263	3186 ± 950	1884 ± 665	4226 ± 1602	53172 ± 14544
V6	56.1 ± 77.4	41781 ± 14395	3271 ± 1127	1939 ± 894	4529 ± 2208	51575 ± 17447

Statistical comparisons within control or nitisinone group denotes whether there were differences across the group visits and to what degree
* <0.05; **<0.01; *** <0.001; **** <0.0001
V1 – V6 refers to visits to study sites at baseline, 3, 12, 24, 36 and 48 months
Values are shown as Mean ± SD

Table S4.

Table S4. 24-h urine metabolites in control (n=69) and nitisinone-treated (n=69) groups in combined female and male group						
	$uHGA_{24}$ µmol/day	$uTYR_{24}$ µmol/day	$uPHE_{24}$ µmol/day	$uHPPA_{24}$ µmol/day	$uHPLA_{24}$ µmol/day	SUM URINE METAB µmol/day
Control group (n=69)						
V1	35387 ± 13868	183 ± 133**	105 ± 86****	72 ± 126	53 ± 73****	36079 ± 13967
V2	31647 ± 18055	216 ± 214	104 ± 77	39 ± 74	55 ± 85	32303 ± 18578
V3	29434 ± 10402	153 ± 110	88 ± 57	99 ± 247	43 ± 79	29906 ± 10795
V4	29895 ± 9354	156 ± 106	73 ± 41	47 ± 126	60 ± 70	30634 ± 9375
V5	33004 ± 11414	147 ± 88	67 ± 40	128 ± 404	133 ± 142	33133 ± 11576
V6	33195 ± 10164	124 ± 74	62 ± 30	173 ± 688	105 ± 138	33866 ± 10236
Nitisinone group (n=69)						
V1	34985 ± 13114****	162 ± 88****	121 ± 270*	44 ± 102****	43 ± 66****	35355 ± 13262***
V2	165 ± 172	1651 ± 1099	75 ± 48	21058 ± 1302)	16183 ± 8748	40349 ± 22252
V3	181 ± 401	1291 ± 662	57 ± 26	15846 ± 5086	13257 ± 4029	30632 ± 9053
V4	317 ± 682	1210 ± 614	54 ± 26	14962 ± 4886	14851 ± 4823	31243 ± 8768
V5	235 ± 364	1320 ± 672	68 ± 48	15163 ± 6115	14653 ± 4997	32643 ± 10075
V6	396 ± 921	1148 ± 635	59 ± 30	14873 ± 6575	12823 ± 6227	30690 ± 11830

Statistical comparisons within control or nitisinone group denotes whether there were differences across the group visits and to what degree
* <0.05; **<0.01; *** <0.001; **** <0.0001
V1 – V6 refers to visits to study sites at baseline, 3, 12, 24, 36 and 48 months
Values are shown as Mean ± SD

Table S5.

Table S5. SUM METAB in control (n=69) and nitisinone-treated (n=69) groups in combined female and male group						
	SUM[¥] HGA µmol	SUM TYR µmol	SUM PHE µmol	SUM HPPA µmol	SUM HPLA µmol	SUM METAB µmol
Control group (n=69)						
V1	36652 ± 14057	3131 ± 1264	2662 ± 899*	75 ± 128	54 ± 74****	42716 ± 15212
V2	32929 ± 18189	3170 ± 1497	2635 ± 766	41 ± 75	56 ± 86	38829 ± 19257
V3	30756 ± 10745	3026 ± 1485	2561 ± 930	100 ± 248	44 ± 79	36485 ± 12065
V4	31341 ± 9663	3141 ± 1166	2913 ± 953	49 ± 127	60 ± 70	37503 ± 10428
V5	34263 ± 12384	3200 ± 1067	2980 ± 792	137 ± 418	133 ± 142	40699 ± 13268
V6	34833 ± 10586	2954 ± 1215	2956 ± 1046	176 ± 694	107 ± 138	41020 ± 11724
Nitisinone group (n=69)						
V1	36361 ± 13330***	3125 ± 977****	2696 ± 901***	45 ± 102****	43 ± 66****	42268 ± 14177****
V2	199 ± 194	45037 ± 13441	2788 ± 877	22876 ± 13301	20246 ± 9416	91145 ± 29629
V3	216 ± 430	44264 ± 13232	2816 ± 817	17552 ± 5285	17356 ± 4829	82204 ± 20743
V4	360 ± 700	43013 ± 14012	3080 ± 1037	16797 ± 5211	19093 ± 5735	82343 ± 21724
V5	277 ± 396	45153 ± 12643	3254 ± 964	17047 ± 6492	18879 ± 5890	84610 ± 22801
V6	452 ± 973	42928 ± 14654	3330 ± 1140	16812 ± 6950	17352 ± 7590	80874 ± 24976

Statistical comparisons within control or nitisinone group denotes whether there were differences across the group visits and to what degree
* <0.05; **<0.01; *** <0.001; **** <0.0001
[¥] SUM refers to TBW + URINE
V1 – V6 refers to visits to study sites at baseline, 3, 12, 24, 36 and 48 months
Values are shown as Mean ± SD

Table S6.

Table S6A. Regression relationships between variables in the control group						
	Age	SUM METAB (µmol)	Change SUM METAB %	cAKUSSI	mAKUSSI	OCH SC
Age (years)		-0.08	0.12*	0.68****	0.59****	0.22****
SUM METAB (µmol)			0.39****	0.09	0.11*	-0.01
Change SUM METAB %				0.12	0.11	0.19**
cAKUSSI					0.95****	0.74****
mAKUSSI						0.56****
OCH SCORES						
**Table S6B. Regression relationships between variables in the NIT group (Females)**						
	Age	SUM METAB (µmol)	Change SUM METAB %	cAKUSSI	mAKUSSI	OCH SC
Age (years)		-0.15	-0.05	0.69****	0.64****	0.63****
SUM METAB (µmol)			0.44****	-0.03	-0.05	-0.02
Change SUM METAB %				-0.03	0.10	-0.07
cAKUSSI					0.93****	0.84****
mAKUSSI						0.65****
OCH SCORES						
**Table S6C. Regression relationships between variables in the NIT group (Males)**						
	Age	SUM METAB (µmol)	Change SUM METAB %	cAKUSSI	mAKUSSI	OCH SC
Age (years)		0.005	0.11	0.77****	0.70****	0.69****
SUM METAB (µmol)			0.42****	0.09	0.12	-0.02
Change SUM METAB %				0.20**	0.16*	0.29***
cAKUSSI					0.96****	0.74****
mAKUSSI						0.56****
OCH SCORES						

Values shown are regression coefficients R
Significance of regression coefficients expressed as: * <0.05; **<0.01; *** <0.001; **** <0.0001

Table S7.

Table S7A. Comparison of variables between the female and male subgroups in the control group (n = 441)		
	Female (n = 179)	Male (n = 262)
Age (years)	48.4 (10.1)	49.2 (9.9)
SUM METAB (µmol)	34353 (12844)****	43499 (14117)
Change SUM METAB %	-5.28 (36.3)	-6.48 (39.3)
cAKUSSI	74.0 (33.3)****	93.3 (34.4)
mAKUSSI	49.9 (24.0)****	64.2 (28.3)
OCH SCORES	18.9 (11.3)	19.2 (10.7)
Table S7B. Comparison of variables between the female and male subgroups in the nitisinone group (n=374)		
	Female (n = 121)	Male (n = 253)
Age (years)	52.9 (8.8)**	49.2 (11.9)
SUM METAB (µmol)	67464 (25099)****	80804 (28612)
Change SUM METAB %	122 (69.4)	111.2 (89.7)
cAKUSSI	80.9 (30.1)**	94.4 (37.1)
mAKUSSI	52.5 (21.1)***	65.4 (30.9)
OCH SCORES	21.6 (11.2)	21.0 (10.5)
Values shown are regression coefficients R<br>Significance of regression coefficients expressed as: * <0.05; **<0.01; *** <0.001; **** <0.0001		

Figure S1A. The phenylalanine/tyrosine pathway

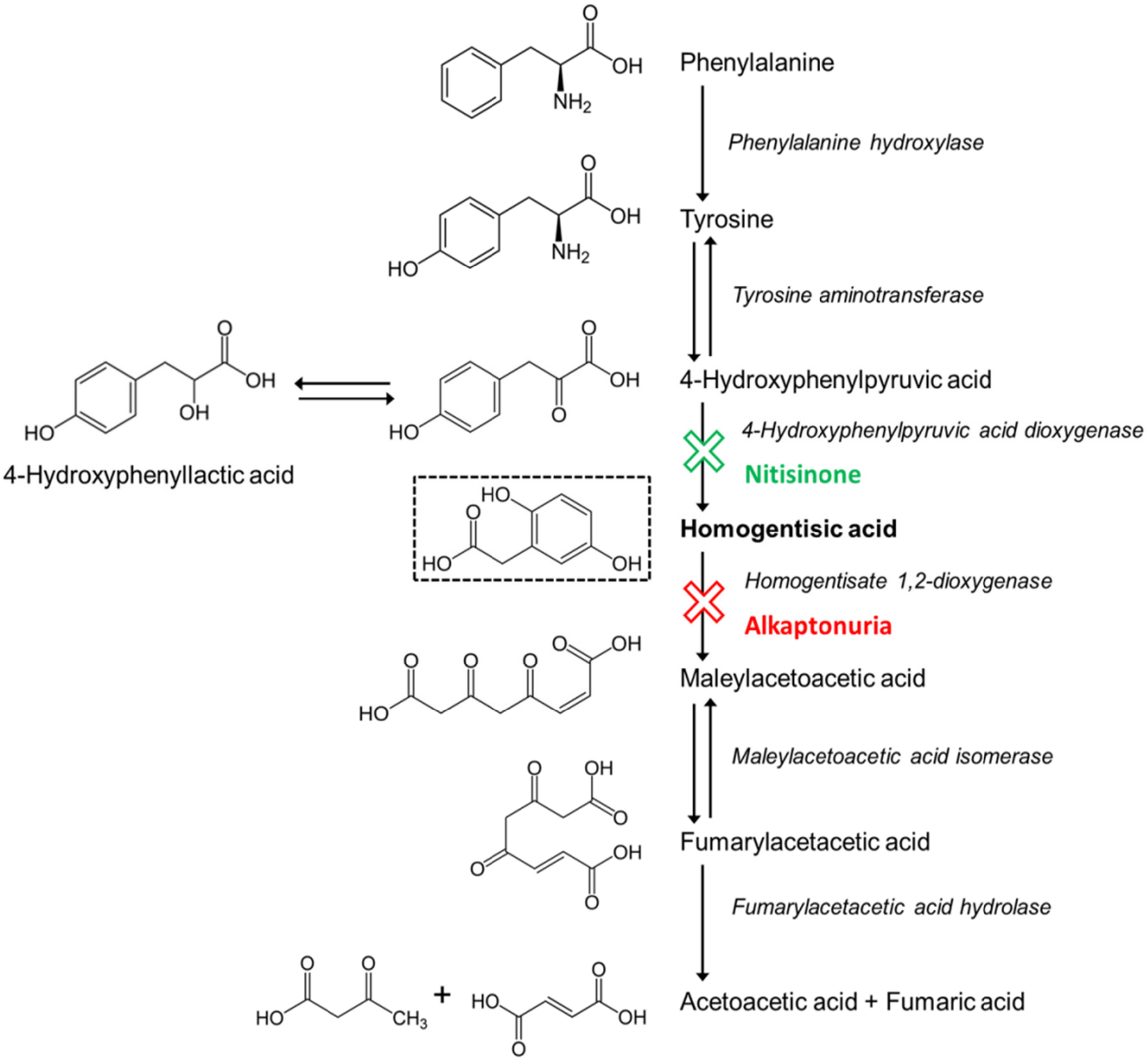

Figure S1B.

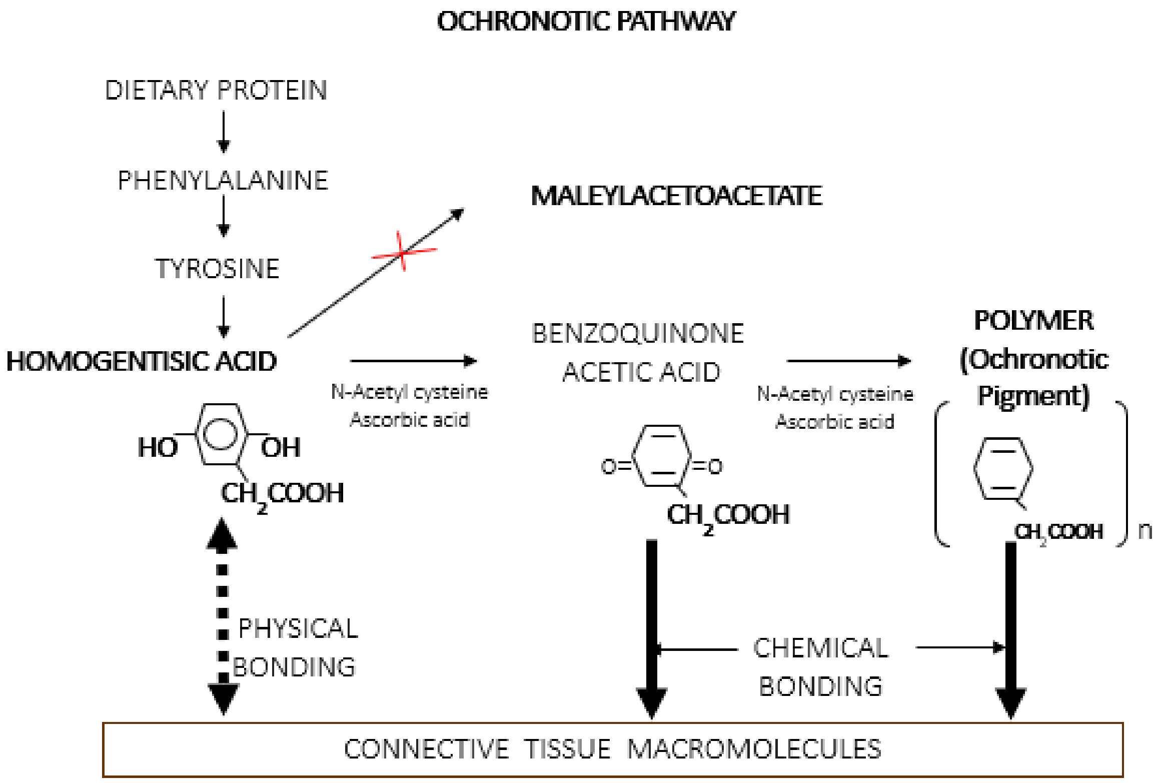

OCHRONOTIC PATHWAY
DIETARY PROTEIN
PHENYLALANINE
TYROSINE
MALEYLACETOACETATE
HOMOGENTISIC ACID
N-Acetyl cysteine
Ascorbic acid
BENZOQUINONE
ACETIC ACID
N-Acetyl cysteine
Ascorbic acid
POLYMER
(Ochronotic
Pigment)
HO
OH
CH2COOH
O=
=O
CH2COOH
CH2COOH
n
PHYSICAL
BONDING
CHEMICAL
BONDING
CONNECTIVE TISSUE MACROMOLECULES

Figure S2.

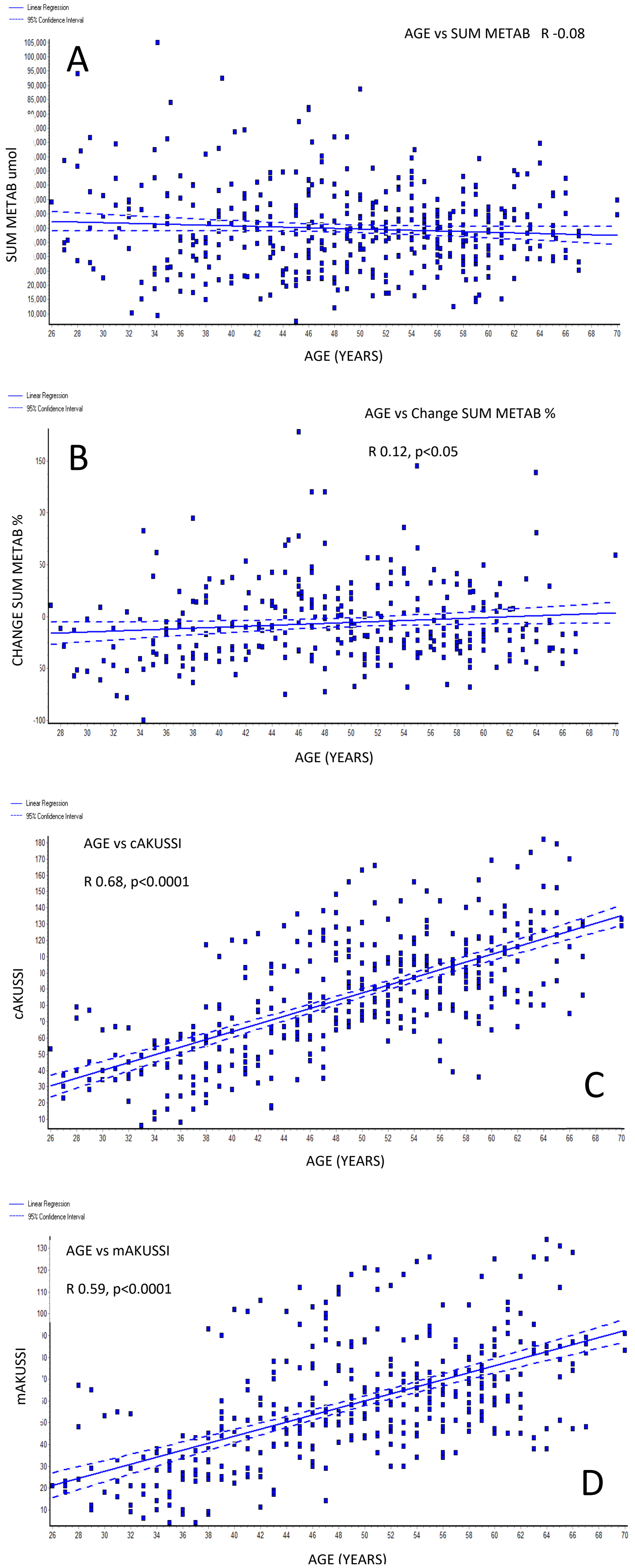
Linear Regression
95% Confidence Interval
AGE vs SUM METAB R -0.08
A
SUM METAB umol
AGE (YEARS)
AGE vs Change SUM METAB %
R 0.12, p<0.05
B
CHANGE SUM METAB %
AGE (YEARS)
AGE vs cAKUSSI
R 0.68, p<0.0001
C
cAKUSSI
AGE (YEARS)
AGE vs mAKUSSI
R 0.59, p<0.0001
D
mAKUSSI
AGE (YEARS)

Figure S3.

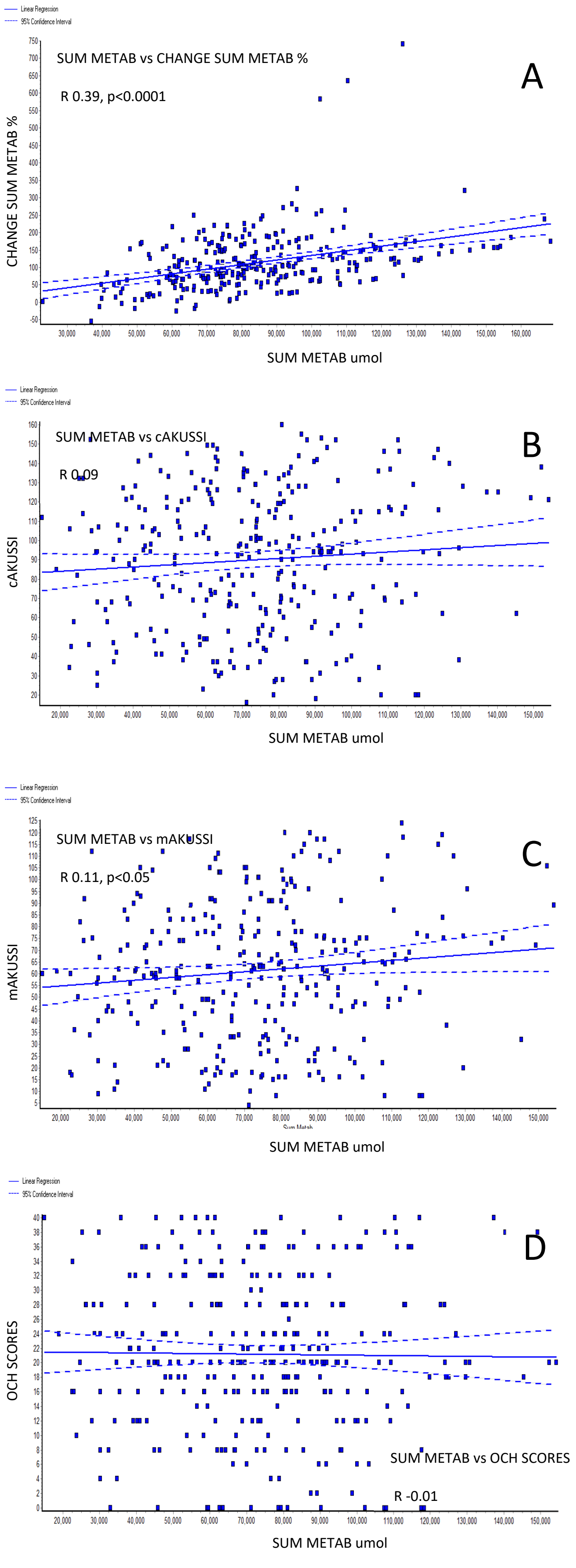
Linear Regression
95% Confidence Interval
SUM METAB vs CHANGE SUM METAB %
R 0.39, p<0.0001
A
CHANGE SUM METAB %
SUM METAB umol
SUM METAB vs cAKUSSI
R 0.09
B
cAKUSSI
SUM METAB umol
SUM METAB vs mAKUSSI
R 0.11, p<0.05
C
mAKUSSI
SUM METAB umol
D
OCH SCORES
SUM METAB vs OCH SCORES
R -0.01
SUM METAB umol

Figure S4.

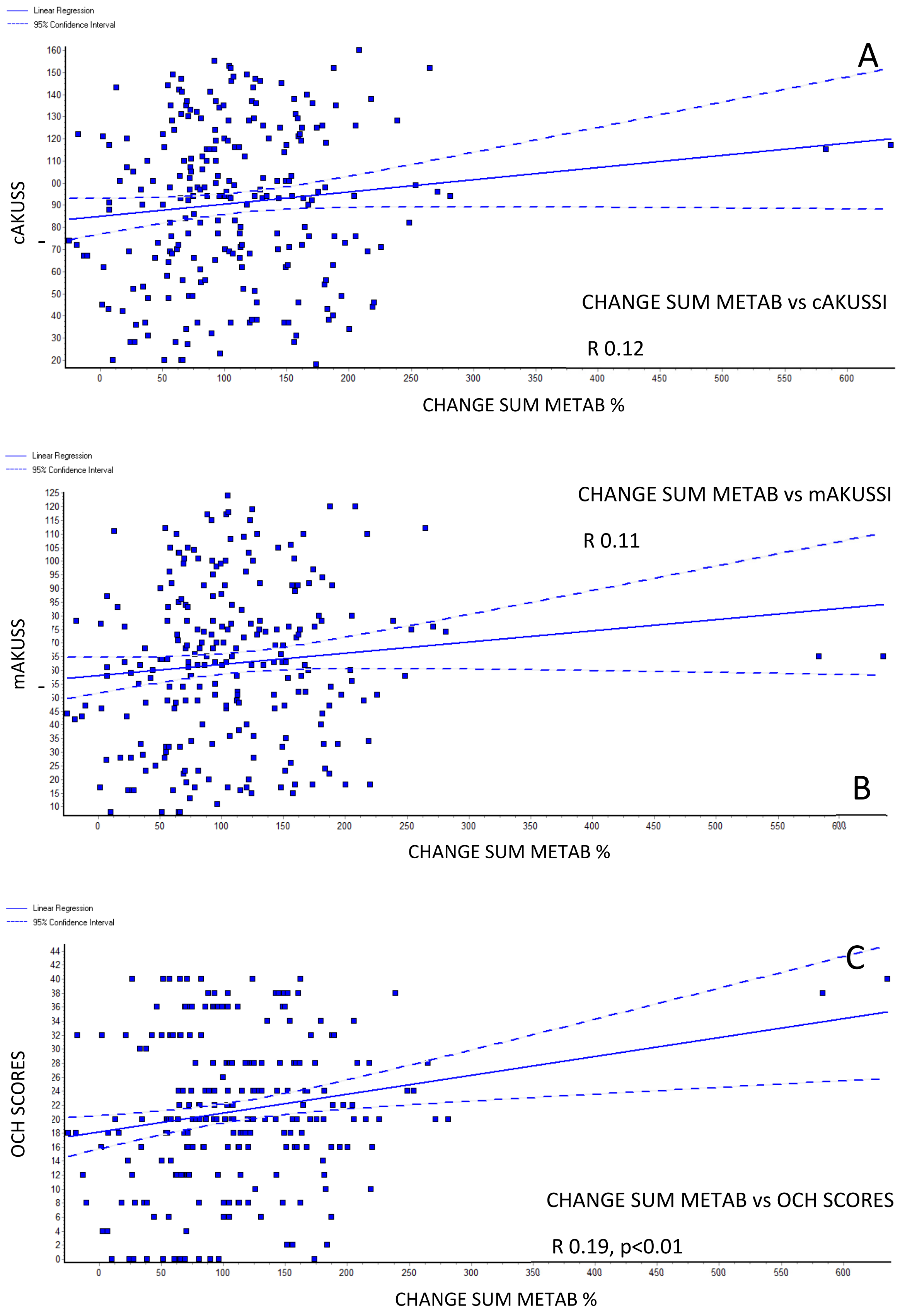

Linear Regression
95% Confidence Interval
A
cAKUSS
CHANGE SUM METAB vs cAKUSSI
R 0.12
CHANGE SUM METAB %
Linear Regression
95% Confidence Interval
CHANGE SUM METAB vs mAKUSSI
R 0.11
mAKUSS
B
CHANGE SUM METAB %
Linear Regression
95% Confidence Interval
C
OCH SCORES
CHANGE SUM METAB vs OCH SCORES
R 0.19, p<0.01
CHANGE SUM METAB %

Figure S5.

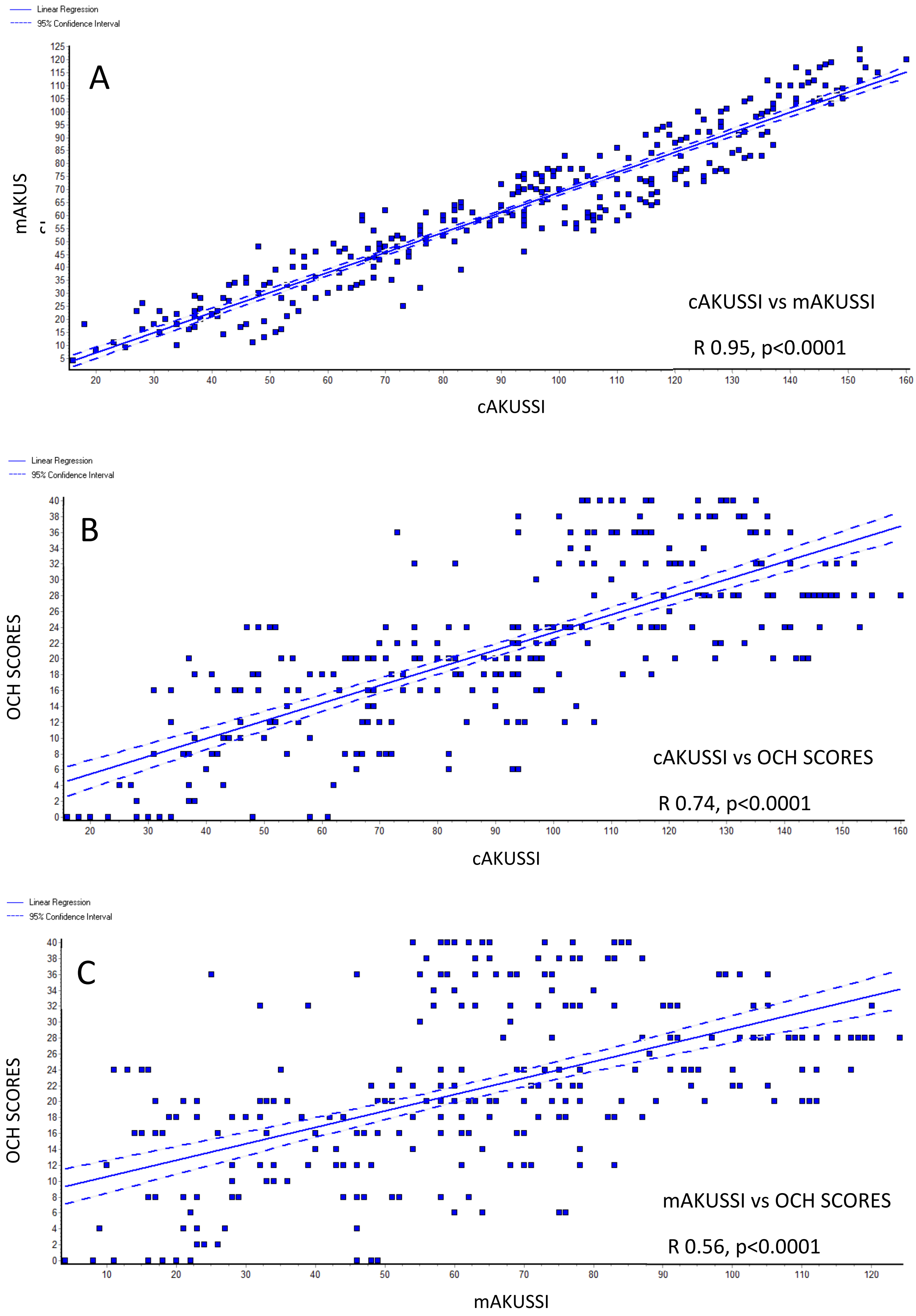
Linear Regression
95% Confidence Interval
A
cAKUSSI vs mAKUSSI
R 0.95, p<0.0001
cAKUSSI
Linear Regression
95% Confidence Interval
B
OCH SCORES
cAKUSSI vs OCH SCORES
R 0.74, p<0.0001
cAKUSSI
Linear Regression
95% Confidence Interval
C
OCH SCORES
mAKUSSI vs OCH SCORES
R 0.56, p<0.0001
mAKUSSI
